# Single-textured insole for the less affected leg in freezing of gait: A hypothesis

**DOI:** 10.3389/fneur.2022.892492

**Published:** 2022-11-30

**Authors:** Mohammad Etoom, Thamer Ahmad Altaim, Anoud Alawneh, Yazan Aljuhini, Fahad Salam Alanazi, Riziq Allah Mustafa Gaowgzeh, Abdullah Owaid Alanazi, Ziyad Neamatallah, Saad Alfawaz, Auwal Abdullahi

**Affiliations:** ^1^Physical Therapy Department, Aqaba University of Technology, Aqaba, Jordan; ^2^Department of Physical Therapy and Health Rehabilitation, College of Applied Medical Sciences, Jouf University, Sakaka, Saudi Arabia; ^3^Department of Physical Therapy, Faculty of Medical Rehabilitation Sciences, King Abdulaziz University, Jeddah, Saudi Arabia; ^4^Medical Rehabilitation Centre, Gurayat General Hospital, Saudi Ministry of Health (MOH), Gurayat, Saudi Arabia; ^5^Department of Physiotherapy, Bayero University Kano, Kano, Nigeria

**Keywords:** asymmetry, freezing of gait, Parkinson's disease, textured insole, proprioception, rehabilitation

## Abstract

Freezing of gait (FoG) is one of the most widely distributed and disabling gait phenomena in people with Parkinson's disease (PD). The current therapeutic interventions show suboptimal efficacy in FoG. Lower extremity proprioception impairments, especially in the most affected leg, gait initiation hesitation, and gait asymmetry are FoG factors, and there is a need to accurately consider them in terms of therapeutic approaches. Accordingly, we hypothesize that using a single-textured insole for the less affected leg may improve FoG by providing proprioceptive stimulation that enhances sensory processing and reduces gait hesitation and asymmetry. Proprioceptive sensory stimulation for the less affected limb could be more effective than for the double legs that are currently used in rehabilitation settings due to the sensory processing in the less affected basal ganglia being better.

## Introduction

Parkinson's disease (PD) is a chronic, progressive neurodegenerative disease characterized by motor and non-motor features (1). One of the motor features is freezing of gait (FoG), which is defined as “brief, episodic absence or marked reduction of forward progression of the feet despite the intention to walk” (2). FoG is one of the most disturbing and disabling gait phenomena in people with PD (2). The overall prevalence of FOG is estimated to be ~40% of patients with PD (3). Although FoG is more common in advanced PD stages, about 28% of patients with PD report FoG in the early stages (3).

Freezing of gait is characterized by trembling of the legs resulting from attempts to overcome freezing and the inability to step forward (2). It typically occurs when patients initiate gait, while turning or dual-tasking, or in changing conditions, such as crossing doorways, narrow areas, or uneven surfaces. Patients with FOG suddenly feel as if their feet are glued to the ground when they try to move forward and are unable to generate any practical steps (3). Most FOG episodes last <10 s, but a few last more than 30 s (4). FOG phenotypes are based on leg movement, which includes shuffling with small steps, leg trembling, and akinesia (5). FOG highly impairs body structure and function, as posture and pain, respectively (6, 7), limits activity as balanced mobility and restricts participation in daily living that worsens the quality of life (4). Furthermore, FoG is a major risk factor for falls in PD (8).

## FoG factors: Proprioceptive impairments, gait initiation hesitation, and gait asymmetry

Freezing of gait is multifactorial and associated with abnormal sensory, cognitive, and motor processing (4). Sensory impairments in the lower limbs, mainly proprioception, contribute to FoG (9). Neuroimaging studies found structural abnormalities in frontoparietal lobes that result in sensorimotor abnormalities that may reflect in FoG (10). People with PD have reduced peripheral sensation arising from the degeneration of cutaneous receptors and peripheral sensory nerves (11). Sensorimotor dysfunction due to proprioceptive losses has also been postulated to induce freezing episodes, where the absence of visual feedback prevents the ability to override faulty proprioceptive feedback (12). Gait initiation hesitation is commonly observed in FoG (2). The hesitation in foot selection to initiate the gait is accompanied by defects in postural adjustments and step onset delay that exacerbates FoG (13, 14).

The gait asymmetry is another FoG factor (15) that arises from asymmetry in basal ganglia degeneration (16). Although FoG is an axial symptom and is often associated with bilateral disease progression, there is clear evidence that confirms the role of gait asymmetry in FoG. The more affected lower limb displayed worse proprioceptive capacity compared with the less affected limb (17) in FoG. Sensory-processing errors in the most affected leg lead to more abnormality in weight distribution, and gait asymmetry and hesitation, and therefore worsen FoG (12). Gait asymmetry could increase the gait coordination complexity that is considered a predictor of FoG according to the threshold model of FoG (18, 19). In sum, proprioception impairments in the most affected leg, gait initiation hesitation, and gait asymmetry are FoG factors.

## FoG management: The need to address FoG factors

Freezing of gait management is challenging due to its multifaceted pathophysiology, and the current evidence shows suboptimal efficacy of the therapeutic approaches (20–22). The suboptimal efficacy raises the attention to address FoG factors in management approaches (4). FoG management requires careful consideration of proprioceptive feedback accuracy, gait asymmetry, and gait initiation hesitation. Therefore, sensory interventions targeting the less affected limb alleviate FoG episodes due to better sensory processing in the less affected basal ganglia compared with the most affected (11). One of the proprioceptive interventions is textured insoles. Textured insoles' intervention provides planter proprioceptive stimulation for single or both legs by enhancing sensory feedback that improves gait and motor performance (23).

## Hypothesis

We hypothesize that using a single-textured insole for the less affected leg may improve FoG. The proprioceptive stimulation to the less affected leg by a single-textured insole may provide better sensory processing, reduce the gait hesitation and gait asymmetry, and increase the attentional focus of the lower legs. Hence, accurate proprioceptive stimulation provided by a single-textured insole may be an effective strategy for alleviating FOG.

## Evaluation of hypothesis

In PD, the failure to translate proprioceptive information into voluntary and reflexive coordinated movements is integral to FoG (24). There is clear evidence that the use of a single-textured insole improves gait asymmetry in healthy individuals and patients with stroke (25–27). The regular use of textured insoles improves plantar sensation, proprioception, and stride length in healthy people and patients with PD (23). The use of the less affected limb is due to the fact that the most affected basal ganglia and limb exhibit more proprioceptive defects and sensory-processing errors in PD and FoG (17). The sensory interventions targeting the less affected limb show a more beneficial effect on FoG (12).

The effect of single-textured insoles can be experimentally examined on FoG episodes and the severity at different conditions and environmental factors, such as multitasking, on and off dopaminergic medication, and different gait speeds. Motor performance, turning time, gait parameters, and reaction time can also be tested to examine the effectiveness of the single texture insole on FoG. The single-textured insole can be tested alone or in combination with other interventions to find the best way to apply it. The intervention can be tested as a potential preventive strategy for FoG.

## Consequences of the hypothesis and discussion

Sensory perception and processing impairment are common in PD and FoG and highly correlated with motor impairments (11). The enhancement of plantar sensory information by textured insoles has been shown to alter gait patterns and improve walking Stability in PD through alternations in spatiotemporal gait parameters (i.e., reduced base of support), sensorimotor function, and gait kinetics and kinematics (28). Therefore, different types of sensory interventions target the motor impairments in PD with careful consideration of the features and factors of motor impairment.

Textured insoles are designed to enhance the somatosensory input of the foot through cost-effective, easy, and accessible passive intervention that can be used during various daily living activities compared with other FoG interventions (29). For example, cue interventions that are widely used in FoG demand high cognitive load and show difficulties in delivering in clinical settings (30). It is recommended to wear a textured insole for long periods to prolong the retention of benefits (23). To overcome the potential sensory habituation, different textures of insoles, in terms of density and location of elevation, i.e., first or second half, can be used interchangeably. We think that a single-textured insole may be more beneficial for the less affected limb in advanced stages of PD and FoG, whichexhibit more asymmetrical PD patterns. Gait initiation by the less affected leg using a single-textured insole may have a better effect.

## Data availability statement

The original contributions presented in the study are included in the article/supplementary material, further inquiries can be directed to the corresponding author.

## Author contributions

ME and YA: state the hypothesis and wrote the draft manuscript. ME, TA, AAlaw, FA, RG, ZN, and SA: writing and reviewing the manuscript. All authors contributed to the article and approved the submitted version.

## Conflict of interest

The authors declare that the research was conducted in the absence of any commercial or financial relationships that could be construed as a potential conflict of interest. The reviewer FL declared a past co-authorship with the author ME.

## Publisher's note

All claims expressed in this article are solely those of the authors and do not necessarily represent those of their affiliated organizations, or those of the publisher, the editors and the reviewers. Any product that may be evaluated in this article, or claim that may be made by its manufacturer, is not guaranteed or endorsed by the publisher.
